# The vaccination threshold for SARS-CoV-2 depends on the indoor setting and room ventilation

**DOI:** 10.1186/s12879-021-06884-0

**Published:** 2021-11-26

**Authors:** A. Mikszewski, L. Stabile, G. Buonanno, L. Morawska

**Affiliations:** 1 International Laboratory for Air Quality and Health, Queensland University of Technology, 2 George Street, Brisbane, QLD 4001 Australia; 2 CIUS Building Performance Lab, The City University of New York, New York, NY 10001 USA; 3 Department of Civil and Mechanical Engineering, University of Cassino and Southern Lazio, Cassino, FR Italy; 4 Global Centre for Clean Air Research (GCARE), Department of Civil and Environmental Engineering, Faculty of Engineering and Physical Sciences, University of Surrey, Guildford, GU2 7XH UK

**Keywords:** SARS-CoV-2, Airborne transmission, Ventilation, Vaccination

## Abstract

**Background:**

Effective vaccines are now available for SARS-CoV-2 in the 2nd year of the COVID-19 pandemic, but there remains significant uncertainty surrounding the necessary vaccination rate to safely lift occupancy controls in public buildings and return to pre-pandemic norms. The aim of this paper is to estimate setting-specific vaccination thresholds for SARS-CoV-2 to prevent sustained community transmission using classical principles of airborne contagion modeling. We calculated the airborne infection risk in three settings, a classroom, prison cell block, and restaurant, at typical ventilation rates, and then the expected number of infections resulting from this risk at varying percentages of occupant immunity.

**Results:**

We estimate the setting-specific immunity threshold for control of wild-type SARS-CoV-2 to range from a low of 40% for a mechanically ventilation classroom to a high of 85% for a naturally ventilated restaurant.

**Conclusions:**

If vaccination rates are limited to a theoretical minimum of approximately two-thirds of the population, enhanced ventilation above minimum standards for acceptable air quality is needed to reduce the frequency and severity of SARS-CoV-2 superspreading events in high-risk indoor environments.

**Supplementary Information:**

The online version contains supplementary material available at 10.1186/s12879-021-06884-0.

## Background

Control of infectious disease is achieved when outbreaks cannot be sustained, and transmission becomes sporadic in nature. For airborne contagion in shared indoor atmospheres, Wells [1] established that the rate of transmission is inversely proportional to the ventilation rate per susceptible occupant. It then follows that to control airborne contagion we can either increase ventilation, or its equivalent through air filtration or disinfection, or decrease the number of susceptible occupants through vaccination [2]. During the COVID-19 pandemic, lockdowns and occupancy controls have been widely applied to reduce transmission of SARS-CoV-2. These are blunt but effective methods of increasing the ventilation rate per susceptible occupant of indoor spaces. As SARS-CoV-2 vaccines become increasingly available worldwide, the question becomes: at what point is the number of susceptibles in public spaces low enough so that occupancy limitations are no longer necessary to control the spread of the virus?

To address this question, we must consider the primary settings of SARS-CoV-2 transmission. As with other agents of airborne contagion such as *Mycobacterium tuberculosis*, SARS-CoV-2 thrives in congregate living and working spaces with shared air, such as prisons, schools, restaurants, abattoirs, and care homes. The COVID-19 pandemic is also fueled by superspreading events in crowded indoor environments where people vocalize and cannot reliably wear masks. For example, Chang et al. [3] modeled full-service restaurants to produce by far the largest increase in infections upon reopening after lockdown of any non-residential location that people visit. Estimates of necessary vaccination rates for these high-risk community settings should be protective in other microenvironments, and therefore approximate a vaccination threshold to control SARS-CoV-2.

The aim of this paper is to estimate setting-specific vaccination thresholds for the wild-type, original strain of SARS-CoV-2 using classical principles of airborne contagion modeling. We included modeling scenarios for a prison cell block and a full-service restaurant, two settings known to be high risk for SARS-CoV-2 transmission. To compare the vaccination threshold for wild-type SARS-CoV-2 to historical estimates for measles virus, we also included a classroom scenario in our analysis. A secondary aim is to quantify how vaccination and ventilation together reduce the pool of potential infectors in each of the settings by estimating the minimum viral emission rate needed to reproduce infection at varying levels of susceptibility.

## Materials and methods

### Approach and definitions

To develop our estimates, we defined a representative exposure scenario for each of the three settings (classroom, prison, restaurant) involving one infectious occupant in a room of typical geometry. We used an established airborne infection risk model to calculate the individual risk of infection (R) for each susceptible occupant, and the event reproduction number (R_event_) at varying ventilation rates and number of susceptibles. R_event_ is the expected number of new infections arising from a single infectious occupant at an event [4]. This is distinct from the basic reproduction number (R_0_), defined as the average number of new infections resulting from the introduction of a single infectious individual into a fully (100%) susceptible host population [5]. For modeling purposes, we quantified the number of susceptibles as the percent of the total occupants who are susceptible to infection (i.e., not immune from vaccination or prior infection). We use the term area concentration of susceptibles to represent the area of indoor space (square meters [m^2^]) per susceptible occupant. The threshold number of susceptibles and the threshold area concentration of susceptibles occur at a calculated R_event_ of one, above which an average of at least one new case is expected. For each setting we calculated these two threshold values at a mechanical ventilation rate based on American National Standards Institute (ANSI)/American Society of Heating, Refrigerating and Air-Conditioning Engineers (ASHRAE) 62.1 standards for acceptable air quality [6], and at a natural ventilation rate when windows cannot be opened and air exchange results solely from infiltration through the building envelope. We then determined the vaccination threshold as the complement of the threshold number of susceptibles assuming no immunity from prior infection and that vaccination confers complete protection. For comparative purposes, we also calculated the threshold values in each setting at a ventilation rate of 15 L per second per person (L s^−1^ p^−1^), a typical goal for high indoor air quality consistent with EN 15251 Category I criteria for a non low polluting building [7].

### ***Calculation of the event reproduction number (R***_***event***_***)***

We used the Gammaitoni and Nucci [8] equation coupled with a Poisson dose–response model to calculate R_event_ for SARS-CoV-2 in a prototypical classroom, prison cell block, and full-service restaurant. The first step is calculating the probability of infection (P_I_) resulting from each exposure through Eqs. (1–3):

1 $$n\left( {t,ER_{q} } \right) = \frac{{ER_{q} \cdot I}}{IVRR \cdot V} \cdot \left( {1 - e^{ - IVRR \cdot t} } \right)\quad \left( {{\text{quanta m}}^{{ - {3}}} } \right)$$

2 $$D_{q} \left( {ER_{q} } \right) = IR\int_{0}^{T} {n\left( t \right)dt} \quad \left( {{\text{quanta}}} \right)$$

3 $$P_{I} = 1 - e^{{ - D_{q} }} \quad \left( \% \right)$$

where n represents the quanta (infectious dose for 63% of susceptible occupants by droplet nuclei inhalation) concentration in air at time t, ER_q_ is the quanta emission rate (quanta h^−1^), I is the number of infectious occupants (assumed to be only one), V is the volume of the indoor environment considered (m^3^), IVRR (h^−1^) represents the infectious virus removal rate in the space investigated, D_q_ is the dose of quanta inhaled by susceptible occupants, T is the total time of the exposure (h), and P_I_ is the probability of infection of a susceptible occupant. The infectious virus removal rate is the sum of the air exchange rate (AER) via ventilation in units of air changes per hour, the particle deposition on surfaces (k_d_, e.g. via gravitational settling), and the viral inactivation in ambient air (λ).

With all other parameters held constant, the probability of infection calculated in Eq. (3) assumes different values based on ER_q_. To evaluate the individual risk (R) of infection of an exposed susceptible occupant for a given exposure scenario, we then quantify the probability of infection as a function of ER_q_ (P_I_[ER_q_]) and the probability of occurrence of each ER_q_ value (P_ERq_) which can be defined by the probability density function (pdf_ERq_) of ER_q_ assuming a lognormal distribution. Since the probability of infection (P_I_[ER_q_]) and the probability of occurrence P_ERq_ are independent events, R for a given ER_q_, R(ER_q_), can be evaluated as the product of the two terms:

4 $$R\left( {ER_{q} } \right) = P_{I} \left( {ER_{q} } \right) \cdot P_{ERq} \quad \left( \% \right)$$

where P_I_(ER_q_) is the conditional probability of the infection, given a certain ER_q_, and P_ERq_ represents the relative frequency of the specific ER_q_ value. The individual risk (R) of an exposed susceptible occupant is then calculated by integrating the pdf_R_ for all possible ER_q_ values, i.e. summing up the R(ER_q_) values calculated in Eq. (5):

5 $$R = \int_{{ER_{q} }} {R\left( {ER_{q} } \right)dER_{q} } = \int_{{ER_{q} }} {\left( {P_{I} \left( {ER_{q} } \right) \cdot P_{ERq} } \right)dER_{q} } \quad \left( \% \right)$$

Equation (5) represents a numerical solution approximately equaling the average P_I_ that would result from a Monte Carlo simulation randomly sampling ER_q_ from its lognormal distribution. The individual risk R also represents the ratio between the number of new infections and the number of exposed susceptible occupants (S) for a given exposure scenario and considering all possible ER_q_ values from its lognormal distribution for the infectious occupant under investigation. For a single exposure event involving a single infectious occupant, R_event_ is calculated as the product of R and S as in Eq. (6):

6 $$R_{event} = R \cdot S\quad \left( {{\text{infections}}} \right)$$

For a specific event, the threshold number of susceptibles occurs at the value of S where R_event_ equals one (S_threshold_ = 1/R) and is calculated by dividing S_threshold_ by the total room occupancy less the infected occupant. The threshold area concentration of susceptibles is calculated by dividing S_threshold_ by the room area.

### Modeling scenario input parameters

Input parameters for the classroom, prison, and restaurant scenarios are summarized in Table 1. Geometry and default occupancy for the classroom are based on the rooms studied by Wells [1] with an exposure time of 5.5 h representing a single day. The restaurant model encompasses the dining room geometry of the US Department of Energy building prototype for a full-service restaurant, with an exposure time of 1.5 h [9]. The prison model is based on the largest cell block size studied by Hoge et al. [10], which was overcrowded with a median living area of 3.2 m^2^ per inmate. The exposure time for the prison scenario is likely highly variable, but we assume it to be 36 h since inmates share the same airspace for extended time periods and peak infectiousness has been estimated to occur at 2 days before to 1 day after symptom onset [11]. Thus, a 36-h period where infectiousness is at or near peak but without symptoms that would prompt quarantine can be reasonably expected.

**Table 1:** Modeling input and ventilation reference parameters

	Classroom	Prison	Restaurant	Average
Room volume (m^3^)	170	576	640	462
Room area (m^2^)	57	160	213	143
Occupancy (persons)	20	50	100	57
Occupancy (m^2^ person^−1^)	2.8	3.2	2.1	2.7
Exposure time (h)	5.5	36	1.5	14
Infectious occupant activity	Standing, speaking	Resting, oral breathing	Resting, loudly speaking	–
Median ER_q_ log_10_ (quanta h^−1^)	0.41	-0.28	1.2	0.44
Natural ventilation AER (h^−1^)	0.5	0.5	0.5	0.5
Mechanical ventilation AER (h^−1^)	2.6	1.4	3.2	2.4
High air quality AER (h^−1^)	6.4	4.7	8.4	6.5
Natural ventilation (L s^−1^ p^−1^)	1.2	1.6	0.89	1.2
Mechanical ventilation (L s^−1^ p^−1^)	6.1	4.4	5.7	5.4
High air quality ventilation (L s^−1^ p^−1^)	15	15	15	15

The distributions for the quanta emission rate were modified from Buonanno et al. for wild-type SARS-CoV-2 ([12]; see Additional file 1) for standing and speaking for the classroom (assuming the class instructor is the emitting subject), resting and loudly speaking for the restaurant, and resting and oral breathing for the prison, with the log_10_ average ER_q_ values indicated in Table 1 and a log_10_ standard deviation for all distributions of 1.2. All susceptible occupants were assumed to be at rest with an inhalation rate of 0.49 m^3^ h^−1^. We used a deposition rate, k_d_, of 0.24 h^−1^ based on the ratio between the settling velocity of super-micrometer particles (roughly 1.0 × 10^–4^ m s^−1^ [13]) and the height of the emission source (1.5 m). For the SARS-CoV-2 inactivation rate in air, we used a value of 0.63 h^−1^ based on the measurements reported by van Doremalen et al. [14]. For each scenario, we varied the AER from zero to a maximum of six air changes per hour to calculate R and R_event_ at a number of susceptibles ranging from 0 to 100%.

## Results

**Fig. 1:**
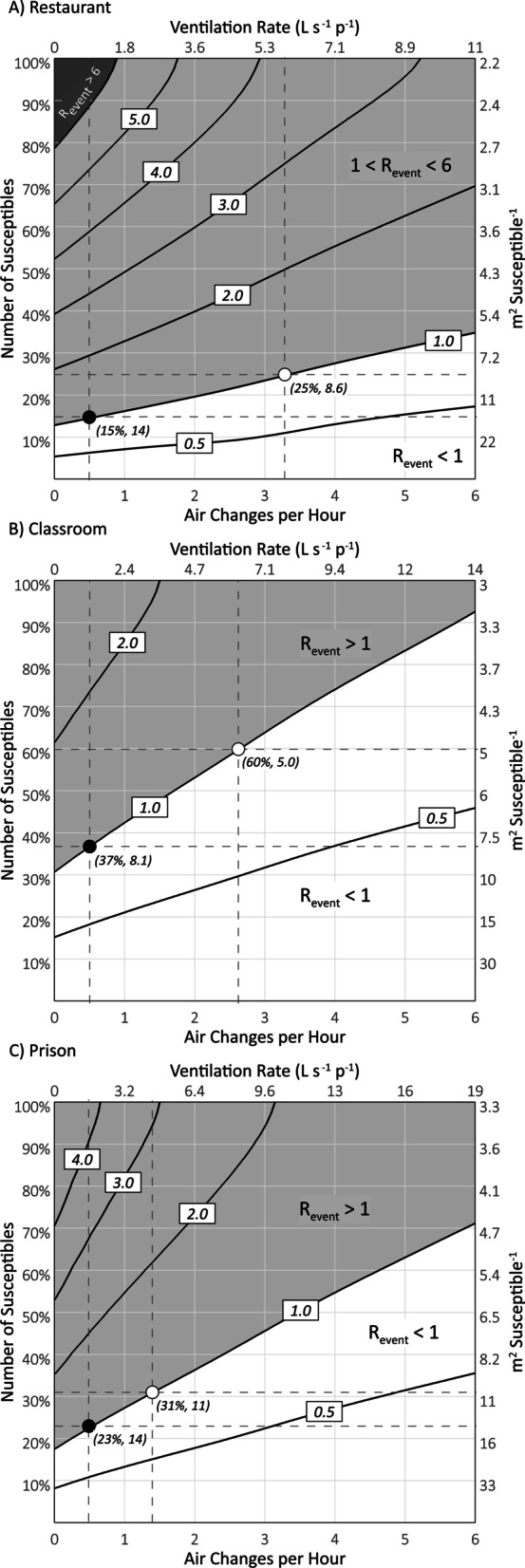
Surface graphs of R_event_ for wild-type SARS-CoV-2 as a function of the number of susceptibles and air exchange rate (AER) for the restaurant (**A**), classroom (**B**) and prison cell block (**C**) modeling scenarios. Contour lines connect equal R_event_ values. The black- and white-filled points along the R_event_ = 1.0 contour line identify the threshold number of susceptibles for natural ventilation and mechanical ventilation scenarios, respectively, at the intersection of the dashed horizontal and vertical lines. The threshold values are labeled in parenthesis in terms of both the percent susceptible and m^2^ susceptible^−1^

The results of our modeling analysis are summarized in Fig. 1 and Table 2 for each setting at an assumed natural ventilation rate of 0.5 air changes per hour, and at a mechanical ventilation rate corresponding to the applicable standard for acceptable air quality based on ANSI/ASHRAE 62.1 and shown in Table 1 [6]. The naturally ventilated restaurant (Fig. 1A) has the lowest threshold number of susceptibles of 15%, and the mechanically ventilated classroom (Fig. 1B) has the highest threshold number of susceptibles of 60%. The threshold number of susceptibles for the prison cell block (Fig. 1C) exhibits the smallest difference between the natural ventilation (23%) and mechanical ventilation (31%) scenarios.

**Table 2:** Modeling results

	Ventilation	Classroom	Prison	Restaurant	Average
	Natural	14%	8.9%	6.8%	9.9%
Individual risk (R) (%)	Mechanical	8.8%	6.5%	4.1%	6.5%
	High air quality	5.5%	3.4%	2.3%	3.7%
Threshold number of susceptibles (%)	Natural	37%	23%	15%	25%
	Mechanical	60%	31%	25%	39%
	High air quality	95%	60%	44%	66%
Threshold area concentration (m^2^ susceptible^−1^)	Natural	8.1	14	14	12
	Mechanical	5.0	11	8.6	8.2
	High air quality	3.1	5.4	4.9	4.5

The average threshold number of susceptibles for all three settings calculated under the natural and mechanical ventilation rates is 32%. In the absence of immunity from prior infections and assuming vaccination confers complete protection, these results suggest an average vaccination threshold of 68% with a range of 40–85%. The naturally ventilated prison and restaurant have the highest threshold area concentration of susceptibles at 14 m^2^ susceptible^−1^, while the mechanically ventilated classroom has the lowest at approximately 5.0 m^2^ susceptible^−1^. The overall average threshold area concentration of susceptibles for mechanical and natural ventilation is approximately 10 m^2^ susceptible^−1^.

Increasing the ventilation rate to the high air quality metric of 15 L s^−1^ p^−1^ increases the threshold number of susceptibles to 95% in the classroom, 60% in the prison, and 44% in the restaurant. The average threshold number of susceptibles for all three settings becomes 66%, more than twice the average of the natural and mechanical ventilation scenarios. To maintain an R_event_ of one in a fully susceptible population, the estimated ventilation requirements are 43 L s^−1^ p^−1^ (24 air changes per hour), 30 L s^−1^ p^−1^ (9.5 air changes per hour) and 17 L s^−1^ p^−1^ (7.0 air changes per hour) for the restaurant, prison, and classroom, respectively. Such high air exchange rates are impracticable in most settings, suggesting a role for ultraviolet air disinfection [15, 16].

**Fig. 2:**
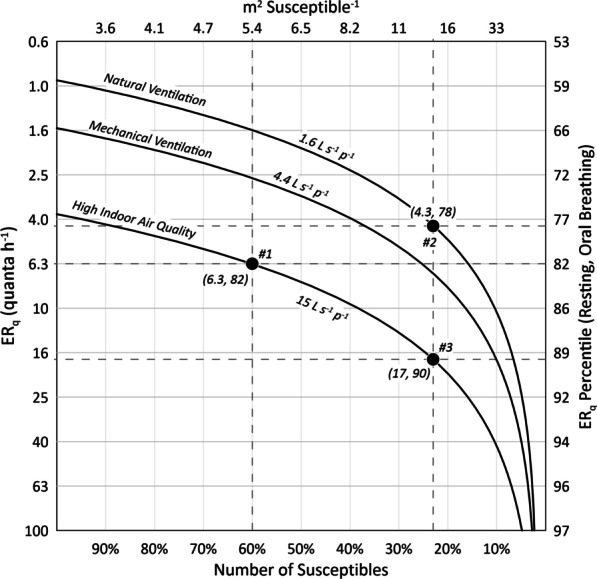
Minimum quanta emission rates (ER_q_) for R_event_ ≥ 1.0 for the prison scenario under natural ventilation, mechanical ventilation, and high air quality ventilation conditions as a function of the number of susceptibles. Points #1 and #2 identify the minimum emission rates for high air quality ventilation and natural ventilation at their respective threshold number of susceptibles from Fig. 1C. Point #3 identifies the minimum emission rate for high air quality ventilation at the natural ventilation threshold number of susceptibles, representing both high ventilation and high vaccination. The minimum emission values are labeled in parenthesis, denoting the emission in quanta h^−1^ and its corresponding percentile in the resting, oral breathing ER_q_ distribution

Increasing ventilation and/or decreasing the number of susceptibles has the effect of increasing the minimum ER_q_ necessary to produce an R_event_ of one, thereby reducing the number of infected occupants capable of infecting others on average. This is illustrated in Fig. 2 for the prison cell block model. For the naturally ventilated cell block in a fully susceptible population, the minimum ER_q_ is just below 1.0 quanta h^−1^, occurring at the 58th percentile value of the resting, oral breathing distribution. At a number of susceptibles of 23%, the minimum ER_q_ becomes approximately 4.3 quanta h^−1^ at the 78th percentile value. Increasing ventilation to 15 L s^−1^ p^−1^ further decreases the pool of potential infectors, raising the minimum ER_q_ to approximately 17 quanta h^−1^ at the 90th percentile value, indicating only a 10% chance of a secondary infection.

## Discussion

The overall average threshold number of susceptibles calculated for the natural and mechanical ventilation scenarios is 32%. This is similar to the threshold inferred from a R_0_ value of approximately 3, as was estimated for the initial wild-type SARS-CoV-2 outbreaks in Wuhan, China and Northern Italy [17]. Our analysis is also consistent with the overdispersed epidemiological nature of SARS-CoV-2 [18], with a minority of cases accounting for most secondary transmissions. In the naturally ventilated prison, we calculate that emissions approximately below the 60th percentile value will fail to reproduce infection, on average, indicating the median emission is not a significant source of transmission (Fig. 2). Furthermore, application of Eq. (5) for the prison scenario shows that emissions above the 80th percentile value account for at least 85% of the total individual risk, suggesting a dispersion parameter (k) between 0.10 and 0.16. This derivation is provided in the Additional file 1 and enables quantification of the probability of SARS-CoV-2 superspreading and outbreak extinction as defined by Lloyd-Smith et al. [19]. Due to this overdispersion, vaccinating 77% of inmates in a naturally ventilated cell block still leaves the remaining susceptible population vulnerable to emitters above the 78th percentile. As a result, explosive but comparatively rare superspreading events may continue in crowded, poorly ventilated settings, a phenomenon that challenges the eradication of measles virus [20]. For reference, exceedance probability plots for the modeled probability of infection results are provided in the Additional file 1 to allow further assessment of this overdispersion.

Applying both high vaccination and high ventilation raises both the threshold number of susceptibles and the minimum emission rate needed to reproduce infection, decreasing the dispersion parameter and increasing the probability of outbreak extinction. Uniformly increasing ventilation to a high air indoor air quality metric of 15 L s^−1^ p^−1^ approximately doubles the average threshold number of susceptibles and therefore halves vaccination requirements for equivalent prevention of infection. Thus, while a ventilation rate of 15 L s^−1^ p^−1^ is unlikely to prevent all secondary infections when a high-emitting index case is introduced into a fully susceptible, indoor population [21], it can provide a substantial downstream epidemiological benefit relative to a poorly ventilated baseline condition. This effect is important for pathogens where transmission is overdispersed, with *M. tuberculosis* being another example [22], as superspreading events (SSEs) facilitate infection of the high-emitting minority that continues the chain of contagion. For our prison cell block model, we estimate that increasing the natural ventilation rate to the high air quality ventilation rate decreases the SSE probability from 16 to 6.6% (see Additional file 1). This is an important finding, as prisons and jails are clear hot spots for SARS-CoV-2 transmission. For example, by March 2021, five California State Prisons (Chuckawalla Valley, California Rehabilitation Center, Avenal, San Quentin, and California Men’s Colony) reported total confirmed COVID-19 case rates above 800 per 1000 inmates [23]. Such high case rates imply a low threshold number of susceptibles, with inadequate ventilation a likely factor. Indeed, during an investigation of the San Quentin State Prison in June 2020, McCoy et al. [24] noted cell blocks with windows that were welded shut and with fan systems that appeared to have been inactive for years.

Historical examples for measles virus illustrating the relationship between ventilation and the threshold number of susceptibles in classrooms are provided by Wells [1, 25] and Thomas [26]. In classic experiments using upper-room air irradiation in primary and upper school classrooms during the 1941 outbreak of measles in suburban Philadelphia, USA, Wells estimated a threshold number of susceptibles of approximately 20% in unirradiated rooms at a then-standard ventilation rate of 14 L s^−1^ p^−1^. Irradiated classrooms supported a much higher threshold number of susceptibles of approximately 57% because the weekly probability of infection in the irradiated rooms was approximately four to five times lower than in the unirradiated rooms [1, 25]. The findings of Wells are similar to those of Thomas [26] who studied the spread of measles in primary schools in the Woolwich district of London in 1904. Thomas concluded that outbreaks of measles tend to occur when the number of susceptibles exceeds approximately 33% and generally continue until the proportion is reduced to 18%. However, the spread of measles in the Woolwich classrooms below the 33% threshold was highly heterogeneous, with many experiencing significant outbreaks infecting a majority of susceptible occupants. The three classes with a number of susceptibles below 10% experienced zero cases of measles, and two temporary schools with crowding and poor ventilation had explosive outbreaks that nearly exhausted the population of susceptibles, with a median probability of infection of 87% for the five classes in the two schools. Thomas measured a carbon dioxide (CO_2_) concentration of 3000 parts per million in one of the temporary schools [26], indicating a steady-state ventilation rate below 2 L s^−1^ p^−1^ and comparable to our natural ventilation scenario. The higher contagiousness of measles as compared to SARS-CoV-2 is illustrated by the historical reported threshold number of susceptibles of 20–33% as compared to our classroom estimate of 37–60% despite the lower ventilation standards of present day. This difference is also reflected by the median classroom measles probability of infection of 87% for the poorly ventilated temporary schools studied by Thomas [26] as compared to the individual risk (R) of approximately 14% we calculated for wild-type SARS-CoV-2 (Table 2). A ventilation rate of 14 L s^−1^ p^−1^ appears sufficient, on average, to prevent sustained airborne transmission of wild-type SARS-CoV-2 in a classroom with a number of susceptibles up to approximately 90%.

The concept of a threshold number of susceptibles for airborne contagion was also evaluated by Kelker [27] (further described by Gorham [28]), based on induced outbreaks of canine distemper virus (CDV) in small ferret populations. CDV is a highly contagious morbillivirus affecting a range of carnivores, and infection of ferrets with CDV is a common animal model for measles pathogenesis [29]. Kelker [27] determined that unless ferrets were densely packed together, with 70% of the population immune (30% number of susceptibles), and with the immune animals spread uniformly throughout the population, outbreaks caused by the introduction of a single CDV-infected ferret had high extinction probability and faded after only a small fraction of susceptibles were infected. In other words, it was difficult to initiate and sustain an outbreak in a population less than 30% susceptible to CDV [28].

## Limitations

A limitation of our infection risk modeling approach is the assumption of a homogeneous concentration of droplet nuclei within the room, with viral emissions being instantaneously and completely mixed. In reality, the risk presented by inhalable virus-laden particles will be substantially higher for a susceptible person directly breathing the respiratory jet in close proximity (generally < 2 m) to an infected host [30, 31], and air speed, local flow direction, and the velocity of the emission itself (for example, a cough versus a normal exhalation) all affect the fate of the jet and the resulting zone of elevated risk relative to the completely mixed condition. While this is a significant limitation, the use of a homogeneous exposure-point concentration is standard practice in environmental risk assessment [32], and it is generally not possible to predict exact locations of infected hosts and their proximity to susceptible occupants of a shared indoor atmosphere, and their relative positions change in time. Similarly, local airflow conditions are complex, variable, and cannot be accurately represented without computational fluid dynamics (CFD) modeling or site-specific tracer testing. Thus, the most defensible approach, also used in environmental risk assessment [32], is to assume susceptible persons are equally exposed to a uniform, average concentration within the exposure unit (the room), fully knowing that some individuals inhale a larger dose than others. From this perspective, the complete and instantaneous mixing of the viral emission into a room is a quite reasonable approach to generate an average, time-variable exposure point concentration. Supporting this, a recent comparison of this box-modeling approach with CFD simulations for a classroom environment indicates relatively minor errors for natural (6%) and forced mechanical (29%) ventilation scenarios [33]. The uncertainty in the emission rate, based on viral loads that vary several orders of magnitude between individuals and over time [34], is likely much more significant than that caused by incomplete mixing at the small scale of our models. For example, the existence of superemitters who generate and emit substantially more particles than the average person, independent of respiratory tract infection, has been demonstrated for breathing [35], the cough [36], and speech [37]. While undoubtedly important, high viral load is not a *sine qua non* for contagiousness, just as sputum smear status is not for *M. tuberculosis* [38]. As such, further improvements to the emission rate distributions are needed that incorporate variation in droplet volume concentrations [34, 39], such that a more complete stochastic emission model can be implemented.

An additional limitation is our estimation of vaccination thresholds using singular, setting-specific events, without considering cumulative exposure effects that may result from an infectious person attending class in 2 successive days, for example. The importance of singular SSEs on SARS-CoV-2 transmission is well established, and such events likely occur during a narrow 1–2 day window of peak infectivity [40]. As such we do not expect cumulative exposures to be a significant factor outside of co-habitation environments, which is why our prison scenario used a 36-h duration. Our approach also does not account for extreme examples such as someone visiting multiple similar restaurants for similar durations on the same evening (thus increasing the number of exposed susceptibles to a similar infectious dose), or for a bartender or other vocalizing restaurant employee who may be present for much longer than 1.5 h. Indeed, there are numerous other scenarios, such as choirs or high-intensity exercise rooms, where higher vaccination thresholds are likely, reinforcing the need for high levels of both vaccination and ventilation also considering that vaccines are not 100% protective.

The emergence of the more transmissible Delta variant in 2021 led to a worldwide resurgence of SARS-CoV-2 and revealed waning efficacy of the first-generation vaccines at preventing clinical infection. A case study [41] of an outbreak in a highly vaccinated (79%) prison illustrates the challenges presented by the Delta variant, particularly in a highly contagious environment such as a prison. Attack rates during the outbreak were as follows:

- 100% for unvaccinated persons without a prior SARS-CoV-2 infection (35 of 35);
- 78% for vaccinated persons without a prior SARS-CoV-2 infection (128 of 164);
- 57% for unvaccinated persons with a prior SARS-CoV-2 infection (4 of 7); and
- 5% for vaccinated persons with a prior SARS-CoV-2 infection (1 of 21).

For this outbreak, a spectrum of susceptibility was observed, with by far the least susceptible group being inmates who were fully vaccinated *and* had a prior infection. This spectrum is further complicated by different attack rates observed depending on the type of vaccine received by the inmates [41]. The threshold number of susceptibles for this outbreak was extremely low, as all fully naïve inmates were infected. A high attack rate for the Delta variant (50%) was also observed in a classroom setting similar to the scenario modeled herein, with the class instructor as the infected host [42]. When considering our model does not include close proximity interactions, the more transmissible Delta variant, and the observed variable vaccine efficacy and resulting spectrum of susceptibility, it is clear that our estimates reflect a lower bound. Furthermore, in light of the extreme contagiousness of the Delta variant, more emphasis should be placed on improving air flow distribution to minimize the formation of stagnation zones, instead of simply increasing airflow in its current configuration [43].

## Conclusions

Our fully prospective airborne infection modeling results are consistent with the transmission dynamics of SARS-CoV-2 and illustrate the challenges presented by substantial heterogeneity in the settings of contagion and a skewed viral emission rate distribution. To support pre-pandemic levels of occupancy, required vaccination rates are much higher for a naturally ventilated restaurant (85%) than for a mechanically ventilated classroom (40%). These estimates reflect a lower bound as they are based on wild-type SARS-CoV-2 and do not consider the more transmissible variants of concern, such as Delta. Regardless, as vaccination campaigns progress around the world, it follows that occupancy limitations should be relaxed for classrooms before full-service indoor restaurants. Maintaining focus on enhanced ventilation together with vaccination is especially important considering the more transmissible Delta variant which is associated with increasing possibility of second infections or vaccine breakthrough infections. Avoidance of overcrowding remains a critical strategy to minimize airborne transmission, as our calculations suggest ensuring an average of 10 m^2^ per susceptible occupant of an indoor space is approximately equivalent to achieving a number of susceptibles of 32% of normal occupancy. This is because the ventilation rate per susceptible occupant is more than tripled relative to the baseline average occupant loading of 2.7 m^2^ per susceptible occupant for the three settings evaluated herein.

## Supplementary Information

**Additional file 1:** Supplementary Material.

## Data Availability

All data generated or analysed during this study are included in this published article and its Additional file 1.
